# Impact of the Semiconductor Defect Density on Solution-Processed Flexible Schottky Barrier Diodes

**DOI:** 10.3390/mi13050800

**Published:** 2022-05-21

**Authors:** Julio C. Tinoco, Samuel A. Hernandez, María de la Luz Olvera, Magali Estrada, Rodolfo García, Andrea G. Martinez-Lopez

**Affiliations:** 1 Micro and Nanotechnology Research Centre (MICRONA), Universidad Veracruzana, Veracruz 94294, Mexico; jutinoco@uv.mx (J.C.T.); samuhernandez@uv.mx (S.A.H.); 2 Facultad de Ingeniería de la Construcción y el Hábitat (FICH), Universidad Veracruzana, Veracruz 94294, Mexico; 3 Solid-State Electronics Section, Electrical Engineering Department, CINVESTAV-IPN, Mexico City 07360, Mexico; molvera@cinvestav.mx (M.d.l.L.O.); mestrada@cinvestav.mx (M.E.); 4 University Center UAEM Ecatepec, Universidad Autónoma del Estado de México, Ecatepec de Morelos 55020, Mexico; rzgarcial@uaemex.mx

**Keywords:** zinc oxide films, solution-processing electronics, Schottky barrier diodes, semiconductor defects

## Abstract

Schottky barrier diodes, developed by low-cost techniques and low temperature processes (LTP-SBD), have gained attention for different kinds of novel applications, including flexible electronic fabrication. This work analyzes the behavior of the *I*–*V* characteristic of solution processed, ZnO Schottky barrier diodes, fabricated at a low temperature. It is shown that the use of standard extraction methods to determine diode parameters in these devices produce significant dispersion of the ideality factor with values from 2.2 to 4.1, as well as a dependence on the diode area without physical meaning. The analysis of simulated *I*–*V* characteristic of LTP-SBD, and its comparison with experimental measurements, confirmed that it is necessary to consider the presence of a density of states (DOS) in the semiconductor gap, to understand specific changes observed in their performance, with respect to standard SBDs. These changes include increased values of *Rs*, as well as its dependence on bias, an important reduction of the diode current and small rectification values (*RR*). Additionally, it is shown that the standard extraction methodologies cannot be used to obtain diode parameters of LTP-SBD, as it is necessary to develop adequate parameter extraction methodologies for them.

## 1. Introduction

During the last decades, the microelectronics industry has been exploring a technological diversification, which allows the possibility of developing specific electronic systems for nontraditional areas, like medical and health care systems, environmental, biological applications, detection systems for chemical or physical signals, etc. Further development of novel materials and fabrication methodologies is required to produce new devices with the desired features. Some of these applications can require substrates, like flexible, transparent, organic, paper, among others [1]. In this context, film deposition from precursor solutions appears to be a potential tool for novel materials and electronic device fabrication techniques [2,3].

On another hand, Schottky barrier diodes (SBD), based on nanostructured oxide semiconductor films, appear to be potential candidates for different kinds of sensor devices. Furthermore, the possibility of using solution-processing techniques for diode manufacture allows the reduction of the fabrication temperatures to levels which make the full fabrication process compatible with flexible substrates. In recent years, the development of SBD based on solution-processes has become an interesting technological approach for manufacturing flexible and paper-based electronic devices, including a variety of sensor devices.

Up to now, for the analysis of low-cost and low temperature processed SBD, (LTP-SBD), thermionic emission was considered the main conduction mechanism. The diode parameters, such as barrier height (*ϕ_b_*), ideality factor (*η*), and series resistance (*R_S_*), are obtained using parameter extraction methodologies developed for high quality, crystalline semiconductor-based SBD, processed at high temperatures.

In LTP-SBD, three main features have been observed in diode parameters, extracted using above mentioned methodologies [4,5,6,7,8,9,10,11]: (i) the rectification ratio (*RR* = *I_ON_*/*I_OFF_*) is, usually, very small (one or two orders of magnitude); (ii) large ideality factor values (*η* > 2), and (iii) relatively large series resistances (*R_S_*). Regarding *η*, values close to 2, even greater than 7, can be found in the literature [4,5,6,7,8,9,10,11]. For SBD processed using high vacuum techniques, like sputtering deposition at room temperature, values of *η* near to 1 have been found [12,13].

Trying to understand the differences observed in the extracted diode parameters for LTP-SBD, with respect to those obtained for standard SBD, different explanations have been considered, among which are the impact of *R_S_*, the presence of different conduction mechanisms, barrier height inhomogeneities, and interfacial states [5,6,7]. However, the physical reasons behind the wide range of values obtained for the LTP-SBD ideality factor are not clear, nor are the strong differences in parameter values observed in different film deposition methods.

In addition to the possible causes of this observed behavior, low temperature processing could jeopardize the semiconductor film quality, since it is well known that noncrystalline materials present a density of localized states (DOS) within the energy gap, which can strongly affect the behavior of devices based on these materials.

In this work, the behavior of the *I–V* characteristic of solution processed, ZnO LTP-SBD is studied. Main diode parameters, obtained by four extraction methods used for standard SBD fabricated at higher temperatures, are analyzed to evaluate the possibility of using them to characterize LTP-SBD. Additionally, simulated *I–V* characteristic of SBD, considering the presence of a density of localized states inside the semiconductor gap, were obtained to analyze the origin of the main characteristics of diodes performance. 

## 2. Experimental Part

### 2.1. Fabrication Process

ZnO Schottky barrier diodes were obtained as follows: (i) the synthesis of ZnO nanoparticles; (ii) the deposition of a film, consisting of the ZnO nanoparticle colloidal dispersion, on a polyethylene terephthalate (PET) substrate, covered by an indium tin oxide (ITO) film as back-side electrode; (iii) deposition, by screen-printing technique, of a top silver electrode. The fabrication process was limited to a maximum temperature of 150 °C. A detailed fabrication process can be found in [11]. Devices with square shape and different length (*L*) were manufactured and then electrically characterized.

### 2.2. SBD Simulation

SBDs were simulated using the ATLAS simulation program from Silvaco [14]. ZnO was considered the semiconductor material and the presence of DOS was included. 

As already mentioned, noncrystalline semiconductor materials contain certain distributions of DOS, which dominate the overall device electrical characteristics. Such states are grouped into deep and tail states. For our study, the effect of the tail states is predominant, so we will only consider them in our simulations. Tail state energy distribution can be approximated to an exponential distribution as:

(1) $$g\left(E\right)=N_{TA}exp\left(-\frac{E_{C}-E}{E_{TA}}\right)+N_{TD}exp\left(-\frac{E-E_{V}}{E_{TD}}\right)$$

where *N_TA_* and *N_TD_* are, respectively, the acceptor and donor density of the tail states at the corresponding band border. *E_TA_* and *E_TD_* are, respectively, the activation energy of the acceptor and donor tails. 

Main material parameters are shown in Table 1. For the DOS, a symmetrical variation for the acceptor and donor tail states was considered, while the value of *N_TA_* and *N_TD_*, was varied from 10^18^ to 10^20^ cm^−3^ eV^−1^. For *E_TA_* and *E_TD_*, typical values for metal oxide materials were considered. Two different carrier densities, *N_B_*, were analyzed. The metal work function (*Φ_M_*) was fixed to produce a barrier height of 0.54 eV.

## 3. Traditional Extraction Methods to Obtain SBD Main Parameters from I–V Curves

Considering that the thermionic emission is the main conduction mechanism in SBD, four *I*–*V* extraction methodologies have been used to determine the diode parameters.

### 3.1. Ideal Extraction Method

The diode current (*I_D_*) of an ideal SBD diode is defined by the general diode equation (GDE) expressed as:

(2) $$I_{D}=I_{0}\left[exp\left(\frac{qV_{D}}{\eta kT}\right)-1\right]\;$$

where *V_D_* is the applied voltage and *η* is the ideality factor.

The term *I*_0_ is the reverse current, which is defined as:

(3) $$I_{0}=AA^{*}T^{2}exp\left(-\frac{q}{kT}\phi_{b}\right)$$

where *A* is the device area, A* is the Richardson constant and *ϕ_b_* is the barrier height formed between the metal and the semiconductor.

There are different procedures to determine the barrier height and the ideality factor. Combining (2) and (3), and considering *V_D_* >> *kT*/*q*, the GDE can be expressed as:

(4) $$ln\left(I_{D}\right)=ln\left(AA^{*}T^{2}\right)-\frac{q}{kT}\phi_{b}+\frac{q}{\eta kT}V_{D}$$

Therefore, the semilogarithmic plot of the forward characteristic exhibits a linear dependence where the slope is related to *η* and the *y*-axis intercept with *ϕ_b_*.

### 3.2. Norde’s Function

This method considers the presence of a resistance in series with an ideal diode (*R_S_*). The method was developed without considering the ideality factor [15]. Afterwards *η* was included into the extraction procedure [16].

Considering the series resistance, the GDE is modified as:

(5) $$I_{D}=I_{0}\left[exp\left(\frac{q\left(V_{D}-I_{D}R_{s}\right)}{\eta kT}\right)-1\right]$$

This method is based on the definition of an *F* function, considering *V_D_* >> *kT*/*q*, as [16]:

(6) $$F\left(V_{D},\gamma \right)=\frac{V_{D}}{\gamma }-\frac{kT}{q}ln\left(\frac{I_{D}}{AA^{*}T^{2}}\right)$$

where *γ* is an arbitrary constant greater that *η*.

For values of *γ* greater than *η*, the *F*(*V_D_*,*γ*) vs. *V_D_* plot presents a minimum at the point (*V*_0_, *F*_0_). That point corresponds with a diode current, of value *I_0_* [16]. Hence, the ideality factor can be determined, considering two different values of *γ* (*γ*_1_ and *γ*_2_) and the corresponding diode current values at the minimum of the *F* function [16]:

(7) $$\eta =\frac{\gamma_{1}I_{02}-\gamma_{2}I_{01}}{I_{02}-I_{01}}$$

The barrier height and the series resistance can be determined as [16]:

(8) $$\phi_{b}=F_{01}+\left(\frac{1}{\eta }-\frac{1}{\gamma_{1}}\right)V_{01}-\frac{kT}{q}\frac{\gamma_{1}-\eta }{\eta }=F_{02}+\left(\frac{1}{\eta }-\frac{1}{\gamma_{2}}\right)V_{02}-\frac{kT}{q}\frac{\gamma_{2}-\eta }{\eta }$$

(9) $$R_{s}=\frac{kT}{q}\frac{\gamma_{1}-\eta }{I_{01}}=\frac{kT}{q}\frac{\gamma_{2}-\eta }{I_{02}}$$

### 3.3. Cheung’s Function

The Cheung´s method allows the determination of the ideality factor, as well as the series resistance and the barrier height [17]. Equation (5) can be rewritten, considering the current density (*J_D_* = *I_D_*/*A*) and *V_D_* >> *kT*/*q*, as:

(10) $$V_{D}=R_{s}AJ_{D}+\eta \phi_{B}+\frac{kT}{q}\eta \cdot ln\left(\frac{J_{D}}{A^{*}T^{2}}\right)$$

The derivative of Equation (10) with respect to the logarithm of the current density is defined as [17]:

(11) $$\frac{dV_{D}}{dln\left(J_{D}\right)}=R_{s}AJ_{D}+\frac{kT}{q}\eta$$

As can be seen, a linear dependence with *J_D_* is present and the ideality factor can be determined from the corresponding *y*-axis intercept [17].

Moreover, an *H* function is defined as [16]:

(12) $$H\left(J_{D}\right)\equiv V_{D}-\frac{kT}{q}\eta \cdot ln\left(\frac{J_{D}}{A^{*}T^{2}}\right)$$

Comparing Equations (10) and (12), the *H* function is expressed as [17]:

(13) $$H\left(J_{D}\right)=R_{s}AJ_{D}+\eta \phi_{B}$$

Therefore, the plot of *H* vs. *J_D_* shows a linear behavior. The slope is related to the series resistance and the *y*-axis intercept to the barrier height.

### 3.4. Forward–Reverse (F–R) Function

This method analyses both the forward as well as reverse *I*–*V* characteristic. According to Equation (3), the reverse current is bias independent. However, solution-processed devices usually exhibit an important variation of the reverse current with the reverse voltage [4,5,6,7,8,9,10,11]. Considering the image force lowering effect and a very thin semiconductor film, the reverse current density (*J_R_*) can be expressed as [11]:

(14) $$J_{R}=A^{*}T^{2}exp\left[-\frac{q}{kT}\left(\phi_{b}-\sqrt{\frac{qV_{R}}{4\pi \varepsilon_{0}k_{d}t}}\right)\right]$$

where *V_R_* is the reverse bias, *k_d_* is the dynamic dielectric constant and *t* is the electrical semiconductor film thickness.

Hence, *ϕ_b_* can be determined through the *y*-axis intercept of the semilogarithmic plot of *J_R_* vs. *V_R_*^1/2^.

Furthermore, the series resistance can be extracted by the voltage derivative, with respect to the diode current of the forward characteristic (*dV_F_*/*dI_F_*), which is expressed as [18]:

(15) $$\frac{dV_{F}}{dI_{F}}={\left(\frac{dI_{F}}{dV_{F}}\right)}^{-1}=R_{S}+\eta \frac{kT}{q}\left(\frac{1}{I_{F}+I_{0}}\right)$$

Therefore, *R_S_* can be extracted from the *y*-axis intercept of the plot of the inverse of the current derivative vs. the 1/(*I_F_* + *I_0_*) term [11]. *I*_0_ is calculated through (3) using the *ϕ_b_* value obtained from (14). Additionally, *η* can be extracted, in an independent manner, from the slope of the same plot [11].

## 4. Results and Discussion

**Figure 1:**
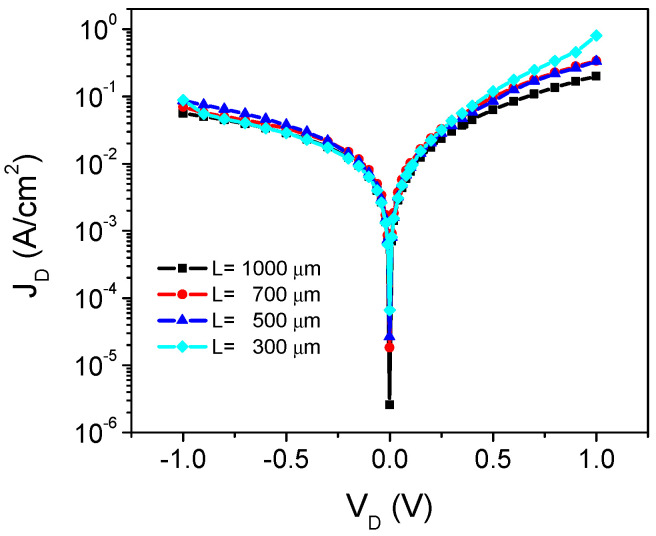
Plot of the *J_D_*–*V_D_* characteristic for the different length devices. The diode area is defined as *A* = *L*^2^.

Figure 1 shows the *J_D_*–*V_D_* characteristics of measured devices with different *L*. As can be seen, the rectification ratio *RR*, obtained as diode current at +1 V divided by the current at −1 V, is about one order of magnitude. Contrarily, for crystalline devices, *RR* can be greater than six orders of magnitude. Moreover, the reverse current density shows a significant dependence on the applied voltage. On the other hand, as expected, the forward current density has similar values for all devices at low applied voltage. Beyond 0.5 V, *J_D_* starts to increase as the device area is reduced. 

Diode parameter extractions, using the different strategies explained in Section 3, were performed with the aim of analysing the diode performance, as well as the unforeseen current density increment, to a deeper level. Table 2 summarizes the extracted values for *ϕ_b_*, *η* and *R_S_*. The overall results agree with the main features previously observed for LTP-SBD [4,5,6,7,8,9,10,11].

**Figure 2:**
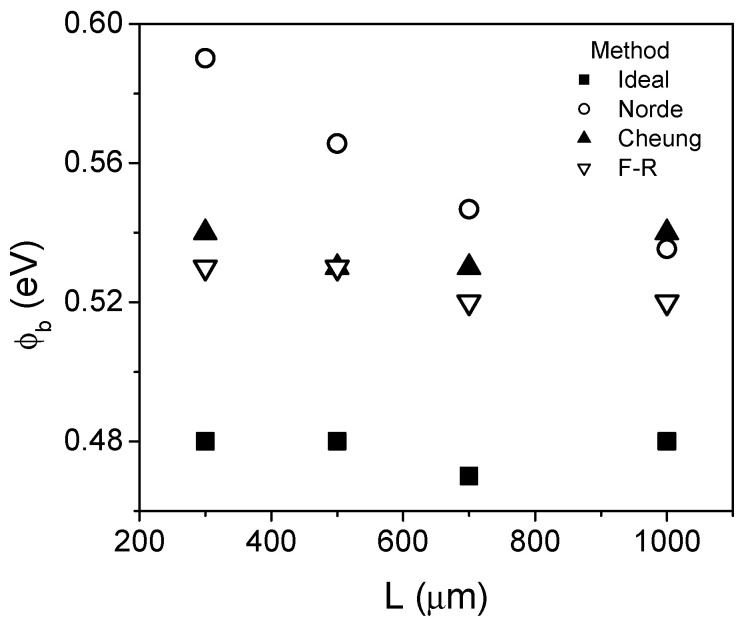
Comparison of the extracted values of the barrier height using the different methods.

Figure 2 shows the comparison of the extracted barrier height obtained with the different methods. As can be seen, for all devices the extracted values of *ϕ_b_* using the ideal method are about 10% smaller than using the other methods. The Cheung and the F–R methods exhibit close values of *ϕ_b_*, while Norde´s method shows a small difference for large devices, which increases as *L* is reduced. Nevertheless, the different methods used allow the determination of the barrier height with a relatively small variation of ±10 %. It is worth noting that the Norde and Cheung methods allow the extraction of the barrier height after the ideality factor; hence, a reliable *η* extraction is of main importance. On the contrary, the F–R method allows the determination of the barrier height in an independent form.

**Figure 3:**
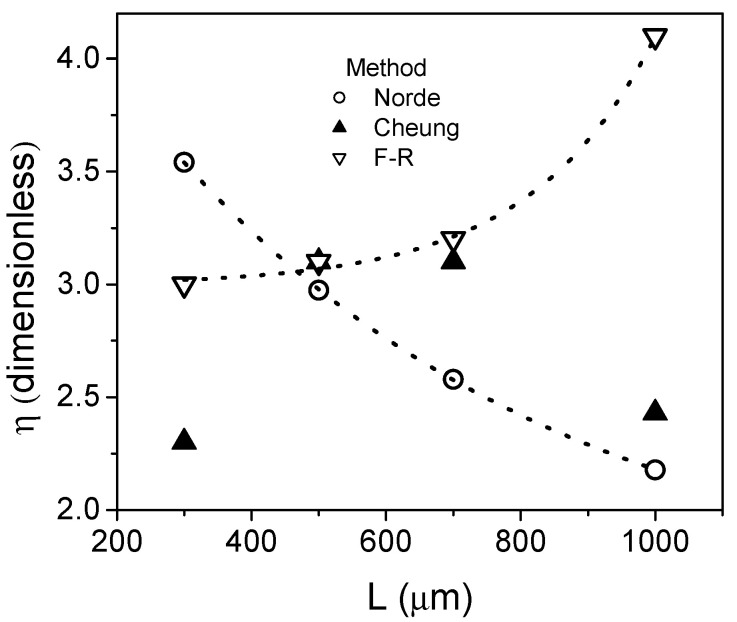
Comparison of the extracted values of the ideality factor.

As can be seen from Table 2, the ideality factor presents abnormally large values when the ideal extraction is used. This occurs because the impact on *R_S_* is neglected. The other methods include the series resistance and, thus, the extracted values of *η* are reduced. Figure 3 shows the comparison of the *η* extracted values using the methods which include the series resistance on the extraction methodology. In all cases, the resulting *η* values are greater than 2 and a significant dispersion is observed. Additionally, Norde and F–R methods show an opposite trend. In the Norde case, the value of *η* increases as the device area is reduced, while with the F–R method, it reduces. However, there is not a physical reason that could support the variation of the diode ideality factor with the device area.

Therefore, the standard SBD extraction methods allow the determination of the barrier height into a reasonable deviation. For the ideality factor, however, a significant dispersion and even different trends when varying the diode area are obtained, depending on the method considered. However, the behavior shown in Figure 1 suggests that a single set of parameters is required to define the diode performance up to ~0.4 V, and after that bias, the increment on the current density with the area must be explained.

**Figure 4:**
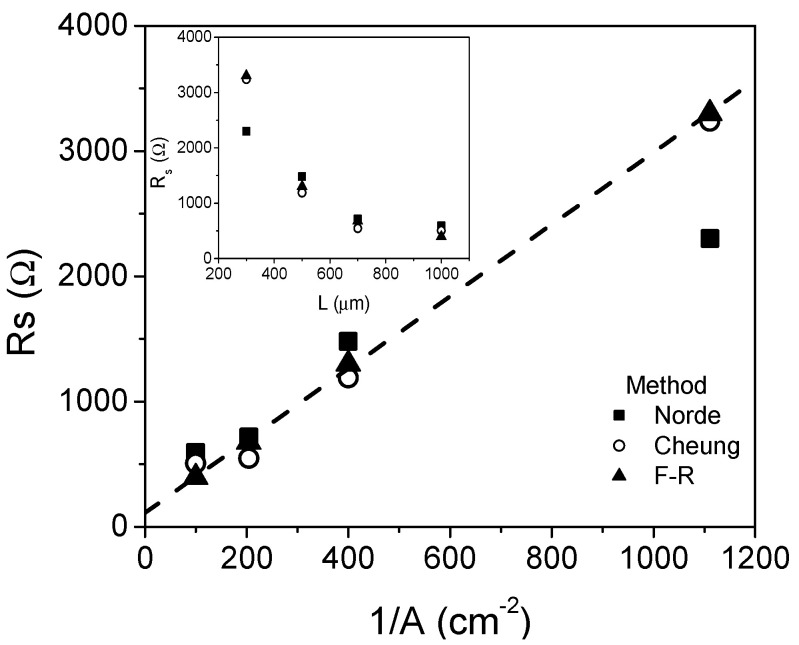
Comparison of the extracted values of the series resistance using the different methods.

Figure 4 shows the extracted *R_S_* vs. the inverse of the diode area. In the inset, the *R_S_* vs. *L* plot is shown. In general, *R_S_* has a linear behavior in respect to 1/*A*. As a first approach, the device can be considered a rectangular semiconductor with electrodes on the top and at the bottom, which produce the observed dependence with the diode area. This fact also suggests a constant value of the resistance normalized with diode area (*A*.*R_S_*). 

The above-mentioned analysis implies an issue on the proper determination of the diode parameters utilizing the traditional methodologies, since a single set of parameters cannot explain the experimental diode current. Because of the observed results, a deeper analysis on the LTP-SBD behavior must be performed, as well as the development of specific extraction methodologies for this kind of devices.

**Figure 5:**
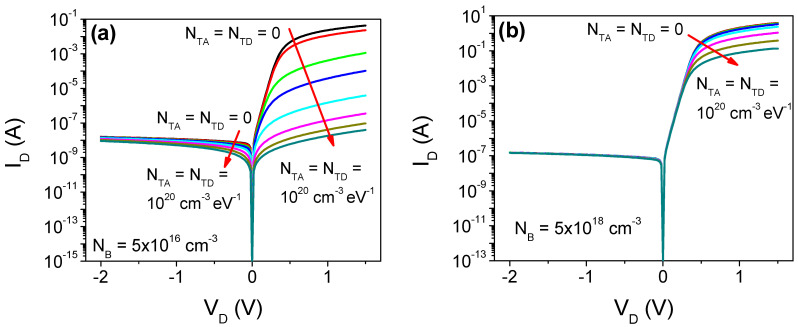
*I*–*V* characteristic for the simulated SBD considering different densities of localized states for *N_B_* equal to (**a**) 5 × 10^16^ and (**b**) 5 × 10^18^ cm^−3^. For comparison, defect free devices (*N_TA_* = *N_TD_* = 0) are considered.

To further analyze the behavior of LTP-SBDs, finite-element numerical simulations were performed. Figure 5a,b show the *I*–*V* characteristic for devices simulated in ATLAS, for two doping concentrations and the different DOS parameters shown in Table 1. For comparison, defect-free simulated devices are included. As can be seen, the reverse current exhibits a negligible impact with the presence of DOS. Contrarily, the tail state’s presence produces an important reduction in the forward diode current. This can be explained due to the electron-trapping on the defects, which implies a reduction in the overall free carriers in the conduction band and, hence, of the device current. The impact, however, is more important for devices with relatively low free carrier concentration, which implies a highly resistive film. In such cases, *I_ON_* is reduced by several orders of magnitude, which explains the typical rectification ratio experimentally achieved [4,5,6,7,8,9,10,11]. 

**Figure 6:**
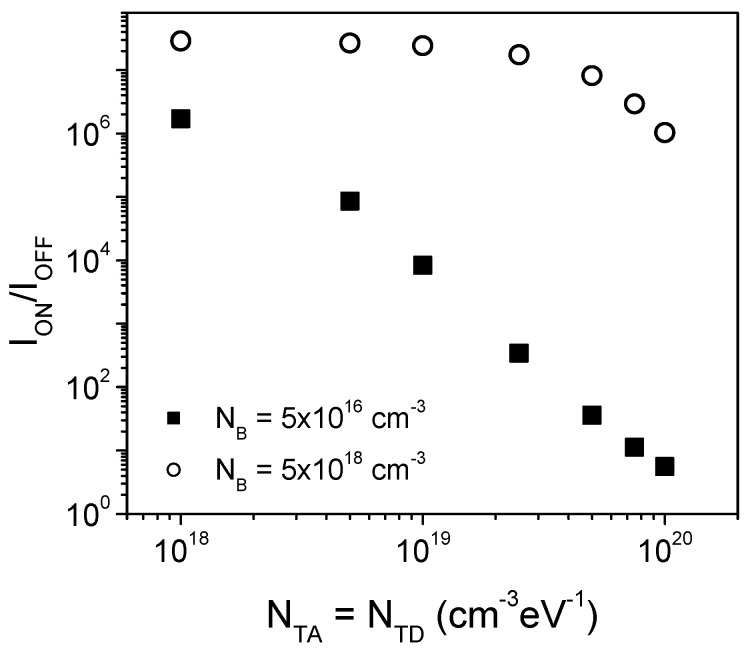
Comparison of the rectification ratio (*RR*) vs. the defect densities, for both *N_B_* values used in the simulations.

Figure 6 shows the comparison of the *RR* vs. the defect densities for two values of *N_B_*. As can be observed, LTP-SBDs with low quality semiconductor films (i.e., high resistivity and high defects densities) exhibit a strong *RR* reduction, until around one order of magnitude, which are similar to what is observed in experimental devices [4,5,6,7,8,9,10,11]. On the other hand, when the layer has a moderate or high conductivity (which implies a better film quality), the impact of the defects is reduced and the *RR* value is only slightly reduced, remaining several orders of magnitude as experimentally observed for high vacuum processing [12,13], even for defect densities in the range of 10^20^ cm^−3^eV^−1^. It can be expected that a further increase of the defect densities will produce a stronger *RR* reduction.

**Figure 7:**
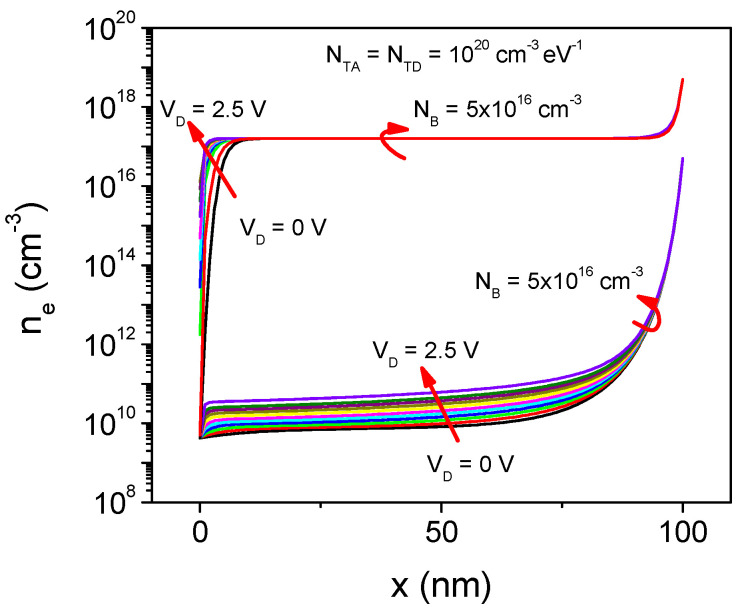
Comparison of the electron concentration (*n_e_*) vs. the semiconductor film position (x), for both *N_B_* values used in the simulations.

Figure 7 shows the simulated electron concentration (*n_e_*) inside the semiconductor film for both *N_B_* values vs. forward applied voltage. As can be seen, the impact of the film qualities on *n_e_* is confirmed. Moreover, it is observed that, for high resistivity films, *n_e_* is modulated by the forward bias. Contrarily, for low resistivity films, *n_e_* is constant along most of the film thickness.

As was mentioned above, the diode can be considered as a rectangular semiconductor die. Therefore, the resistance due to a differential film thickness is defined as:

(16) $$dR_{S}=\frac{1}{q\mu A}\cdot \frac{dx}{n_{e}\left(x\right)}$$

where *μ* is the electron mobility and *A* is the device area.

The total resistance, due to the semiconductor film, can be calculated integrating (16) along the film thickness (*t*):

(17) $$R_{S}=\frac{1}{q\mu A}\overset{t}{\underset{0}{\int }}\frac{dx}{n_{e}\left(x\right)}$$

**Figure 8:**
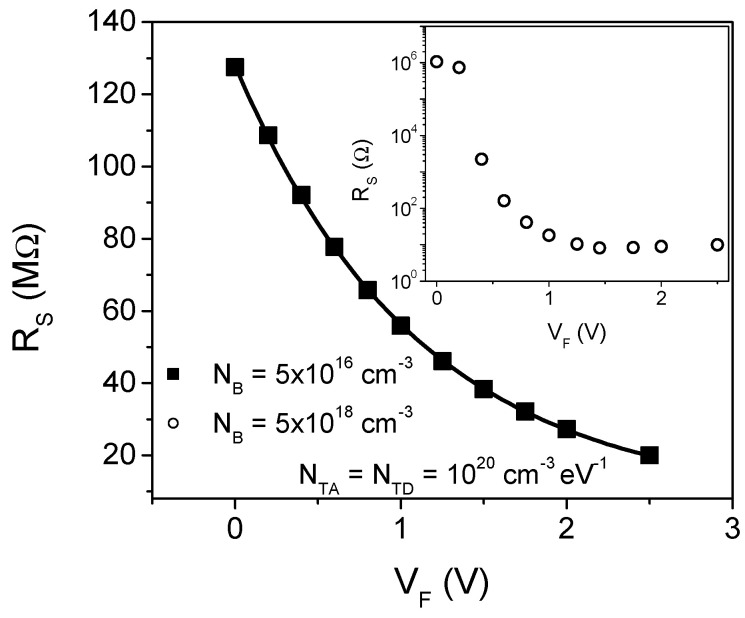
Calculated series resistance vs. forward bias, for *N_B_* of 5 × 10^16^ cm^−3^. In the inset, the corresponding plot for *N_B_* of 5 × 10^18^ cm^−3^ is shown.

Considering the electron distribution shown in Figure 7, it is possible to determine the series resistance contribution caused by the film. Figure 8 shows the calculated resistance vs. the bias applied for device with *N_B_* = 5 × 10^16^ cm^−3^ and defect densities of 10^20^ cm^−3^ eV^−1^. In the inset, the calculated resistance for a device with *N_B_* = 5 × 10^18^ cm^−3^ is also shown. For the case of low resistivity semiconductor film, the resistance shows an abrupt reduction at small forward bias, from about 1 MΩ to few kΩ. When bias is increased above 1 V, *R_S_* becomes almost constant. On the contrary, for a higher resistivity material with a relatively high defect density, the resistance exhibits extremely high values. At the same time, an important dependence on the applied forward voltage is observed. According to Figure 8, *R_S_* exhibits an exponential dependence on *V_D_*, which, as a first approach, can be expressed as:

(18) $$R_{S}=R_{0}exp\left(-\frac{V_{D}}{\delta }\right)$$

where *R_0_* is the zero bias resistance and *δ* can be related to the DOS.

Under this scenario, to better represent the behavior of the *I–V* curve for LTP-SBDs, the general diode equation must be modified as:

(19) $$I_{D}=AA^{*}T^{2}exp\left(-\frac{q\phi_{B}}{kT}\right)exp\left\{\frac{q\left[V_{D}-I_{D}R_{0}exp\left(-\frac{V_{D}}{\delta }\right)\right]}{\eta kT}\right\}$$

This series resistance bias dependence can explain the abnormal current density increment observed as *L* is reduced in Figure 1. As diode area is reduced, the resistance value is increased, and, therefore, its reduction with the applied voltage becomes more significant. Hence, beyond 0.4 V, the current density starts to increase, due to the *R_S_* reduction.

**Figure 9:**
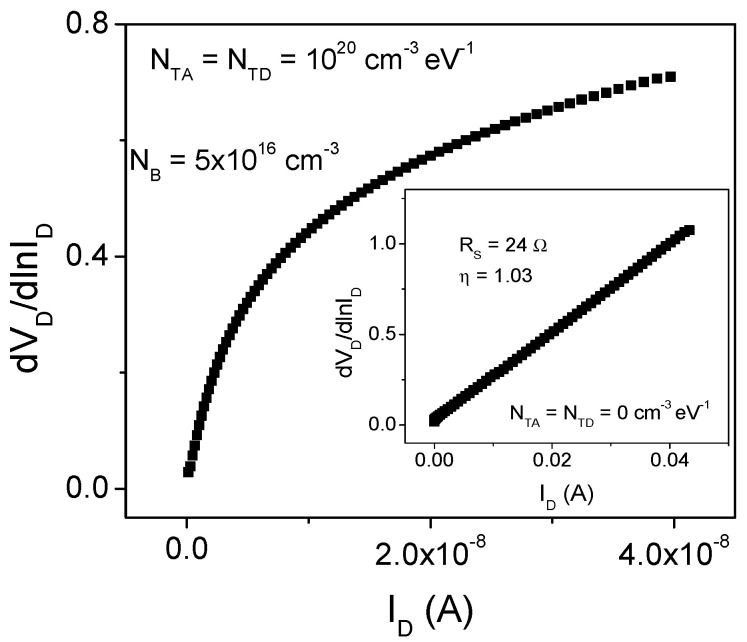
Plot of the *dV_D_*/*dln*(*I_D_*) vs *I_D_*, used for *η* and *R_S_* extraction in the Cheung extraction method.

Furthermore, the bias-dependent *R_S_* would imply an important concern regarding the correct parameter extraction. In order to verify this assumption, Figure 9 shows the corresponding *dV_D_*/*dln*(*I_D_*) vs. *I_D_* plot, according to Equation (11) of Cheung´s method, for the simulated device shown in Figure 8. For comparison, in the inset the plot for a defect free device is shown. As can be seen, the extraction procedure can be properly applied for the defect free device getting the extracted value of *η* as one. On the contrary, when the high defect density is included in the simulation, the plot does not show a linear behavior at any forward bias region. This fact clearly shows that for low-cost and low-temperature processing SBDs, the film quality compromises the reliable application of the traditional extraction methods due to the bias dependence exhibited by *R_S_*. Therefore, the extracted parameters can exhibit the important variations shown in Figure 2 and Figure 3. Thus, proper extraction methodologies for low-cost and low-temperature processed SBD are of main importance to adequately understand the diode behavior.

## 5. Conclusions

ZnO *LTP-SBDs* were analyzed using a single *I_D_*–*V_D_* characteristic and four traditional extraction methodologies. The barrier height extraction shows a relatively small dispersion of about ±10%. On the other hand, the ideality factor obtained exhibits a significant dispersion with values from 2.2 to 4.1, depending on the extraction method used. Simulation results show that devices without or with low DOS, as is the case of standard SBD fabricated at higher temperatures, show high values of RR, relatively small values of *R_S_*, and are almost bias-independent at relatively high forward applied voltage. Thus, traditional parameter extraction methodologies can be properly used. On the other hand, devices fabricated using low-cost techniques (solution-processing techniques, printing strategies, etc.) at low temperatures can produce films with high tail state densities and high resistivity. Simulations showed that the combination of high DOS and low carrier concentration produces a strong impact on diode behavior, which implies an important reduction in the forward current and *RR* values. For these devices, the series resistance exhibits high values, as well as an exponential dependence on the forward applied voltage. Under these conditions, the traditional extraction methodologies of diode parameters are compromised, so further efforts must be made to develop adequate parameter extraction methodologies for low-cost and very low-temperature processed Schottky barrier diodes.

## Figures and Tables

**Table 1:** Summary of the semiconductor film parameters used in simulations.

Parameter	Value	Parameter	Value
*E_g_*	3.2 eV	*N_TA_*, *N_TD_*	10^18^ to 10^20^ eV^−1^ cm^−3^
*χ_s_*	4.3 eV	*E_TA_*	0.105 eV
*N_C_*, *N_V_*	5 × 10^18^ cm^−3^	*E_TD_*	0.385 eV
*μ_e_*	10 cm^2^/Vs	*N_B_*	5 × 10^16^ and 5 × 10^18^ cm^−3^

**Table 2:** Summary of the extracted Schottky barrier diode through the different extraction procedures.

Diode Length (μm)	Ideal Method		Norde’s Function			Cheung’s Method			F–R Method		
	*η*	*ϕ_b_* (eV)	*η*	*ϕ_b_* (eV)	*R_s_* (Ω)	*η*	*ϕ_b_* (eV)	*R_s_* (Ω)	*η*	*ϕ_b_* (eV)	*R_s_* (Ω)
1000	16.5	0.48	2.2	0.53	592	2.4	0.54	506	4.1	0.52	395
700	15.6	0.47	2.6	0.55	714	3.1	0.53	546	3.2	0.52	675
500	14.4	0.48	3.0	0.56	1478	3.1	0.53	1189	3.1	0.52	1300
300	12	0.48	3.5	0.59	2300	2.3	0.54	3238	3	0.53	3300
